# Study of Myocardial Perfusion in Obese Individuals without Known Ischemic Heart Disease

**DOI:** 10.5935/abc.20180250

**Published:** 2019-02

**Authors:** Tufi Dippe Jr., Cláudio Leinig Pereira da Cunha, Rodrigo Julio Cerci, Arnaldo Lafitte Stier Jr., João Vicente Vítola

**Affiliations:** 1 Hospital de Clínicas da Universidade Federal do Paraná, Curitiba, PR - Brazil; 2 Clínica Quanta Diagnóstico e Terapia, Curitiba, PR - Brazil

**Keywords:** Obesity, Diabetes Mellitus, Myocardial Reperfusion/radionuclide imaging, Coronary Artery Disease/physiopathology

## Abstract

**Background:**

Obesity is associated with an increased risk of type 2 diabetes mellitus (DM), ischemic heart disease (IHD) and cardiovascular mortality. Several studies have demonstrated the diagnostic and prognostic value of single photon computed tomography-myocardial perfusion scintigraphy (SPECT-MPI) in the evaluation of patients with suspected IHD, including in obese population. Data on clinical risk factors and their association with abnormal myocardial perfusion in obese patients are scarce in the Brazilian population.

**Objective:**

To determine the factors associated with abnormal myocardial perfusion in obese individuals without known IHD.

**Methods:**

We studied obese patients without known IHD who were referred for evaluation through SPECT-MPI between January 2011 and December 2016. Clinical variables and results of SPECT-MPI were obtained systematically. The distribution of continuous variables was assessed using the Shapiro-Wilk and Shapiro-Francia tests. We used the unpaired Student t test to compare the means of continuous variables with normal distribution and the Chi Square test for binomial variables analysis. A p value < 0.05 was considered statistically significant. The association of the clinical variables for the presence of factors associated with abnormal myocardial perfusion was determined by univariate and multivariate logistic regression analysis, and respective odds ratios (OR) and 95% confidence intervals (CI).

**Results:**

The study sample consisted of 5,526 obese patients. Mean body mass index (BMI) of our patients was 33.9 ± 3.7 kg/m^2^, 31% had DM, and myocardial perfusion abnormalities was observed in 23% of the total sample. The factors associated with abnormal myocardial perfusion on multivariate analysis were: age (OR: 1.02, 95% CI 1.01-1.03, p < 0.001), DM (OR: 1.57, 95% CI 1.31-1.88, p < 0.001), typical angina before the test (OR: 2.45, 95% CI: 1.82-3.31, p < 0.001), need for pharmacologic stress test (OR: 1.61, 95% CI: 1.26-2.07, p < 0.001), less physical effort evaluated in metabolic equivalents (METs) during the exercise treadmill test (OR: 0.89, 95% CI: 0.85-0.94, p < 0.001) and a lower post-stress left ventricular ejection fraction after stress (LVEF; OR: 0.989, 95% CI: 0.984-0.994, p < 0.001).

**Conclusion:**

The factors associated with abnormal myocardial perfusion in obese patients without known IHD were age, DM, presence of typical angina, ventricular dysfunction, and inability to undergo physical stress as clinical variables, in addition to functional capacity during physical stress.

## Introduction

According to the World Health Organization (WHO), obesity is defined as a body mass index (BMI) ≥ 30 kg/m^2^.^1^ In 2016, more than 1.9 billion were overweight, 650 million of them obese.^2^

In Brazil, Vigitel 2016, a nationwide telephone survey of protective and risk factors for chronic diseases, sponsored by the Ministry of Health, revealed that 53.8% of Brazilian adults were above ideal body weight. The proportion of obese individuals older than 18 years was 18.9%.^3^

Obesity is an independent risk factor for cardiovascular disease. Besides, it increases the risk of traditional risk factors, such as systemic arterial hypertension (SAH), type 2 diabetes mellitus (DM) and dyslipidemias, leading to an increased incidence of ischemic heart disease (IHD), cardiovascular mortality and risk of sudden death.^4-6^ Evidence from cohort studies have indicated that obesity is also an independent risk factor for coronary artery disease (CAD).^7-9^

Many studies have shown the diagnostic and prognostic value of single-photon emission computed tomography - myocardial perfusion imaging with (SPECT-MPI) in patients with suspected or confirmed IHD,^10-12^ including obese patients.^13-15^ Nevertheless, information on the predictive role of SPECT-MPI among Brazilian obese subjects are scarce.

The aim of this study was to determine factors associated with abnormal SPECT-MPI in a large population of obese subjects without known IHD.

## Methods

### Patients

Obese patients without known IHD who had undergone SPECT-MPI were studied between January 2011 and December 2016.

The following clinical data were prospective collected using a standardized questionnaire - age, sex, weight, height, BMI, symptoms before the SPECT-MPI test (typical, atypical or no pain, and tiredness), previous heart disease or procedures (coronary cineangiography, acute myocardial infarction, myocardial revascularization surgery and coronary angioplasty), SAH, DM, dyslipidemia, smoking, use of medications and family history of IHD).

Regarding SPECT-MPI, we assessed the type of stress used during the test, treadmill test (TT) alone or combined with pharmacological stress test. Physical exertion during the tests was quantified by metabolic equivalents (METs). We also analyzed myocardial perfusion patterns (normal, ischemia alone or associated with fibrosis), and post-stress left ventricular ejection fraction (LVEF).

All tests were performed using a CardioMD (Philips, Milpitas, CA, USA) or a Vertex (ADAC, Milpitas, CA - USA) gamma camera. All images were reviewed immediately after acquisition, and an additional prone imaging was always obtained when the presence of artifacts was suspected. Both images were considered to define the type of myocardial perfusion defect and the final report also.

### Statistical analysis

All continuous variables are shown as mean and standard deviation, and all categorical variables as absolute values and percentages. Normal distribution of continuous variables was tested by Shapiro-Wilk and Shapiro-Francia tests.

Unpaired Student’s t test was used to compare the means of continuous variables with normal distribution, and the chi-square test used for analysis of binominal variables. A p-value<0.05 was considered statistically significant.

The association of clinical variables, type of the test stress, and left ventricular function with abnormal SPECT-MPI was analyzed by univariate logistic regression, followed by multivariate analysis. The respective odds ratio (OR) and 95% confidence intervals were also calculated.

All analyses were performed using a specific software, the Stata Statistical Software, Release 11 (College Station, TX: StataCorp LP).

## Results

### Demographic characteristics of the patients

From January 2011 to December 2016, a total of 5,526 obese patients were referred for SPECT-MPI. Table 1 shows demographic characteristics of the patients.

**Table 1:** Demographic characteristics of the patients without known ischemic heart disease and body mass index (BMI) ≥ 30kg/m^2^ (n = 5,526)

Characteristics	Mean (standard deviation) or number (percentage)
Age	59.4 (12.2)
BMI (kg/m^2^)	33.9 (3.7)
Male sex	2.605 (47.1%)
Diabetes mellitus	1,727 (31.5%)
Systemic arterial hypertension	4,106 (74.3%)
Family history of IHD	1,081 (19.5%)
Smoking	466 (8.4%)
Dyslipidemia	2,996 (54.2%)
**Symptoms before SPECT-MPI**	
Asymptomatic	2,996 (55.0%)
Atypical angina	1,210 (22.3%)
Typical angina	362 (6.6%)
Tiredness	878 (16.1%)
**Stress protocol**	
Physical	3,576 (64.7%)
Pharmacological	1,950 (35.3%)
Physical exertion, in METs	8.52 (2.37)
LVEF	59.2 (17.6)
LVEF > 50%	4,821 (92.9%)
LVEF 30 - 49%	330 (6.4%)
LVEF < 30%	38 (0.7%)
Abnormal SPECT-MPI	1,288 (23.3%)
Ischemia alone	1,228 (22.2%)
Ischemia > 10% of the LV	74 (1.3%)
Fibrosis alone	22 (0.4%)
Fibrosis and ischemia	38 (0.7%)

BMI: body mass index; IHD: ischemic heart disease; METs: metabolic equivalents; LVEF: post-stress left ventricular ejection fraction; SPECT‑MPI: myocardial perfusion imaging with single-photon emission computed tomography; LV: left ventricle.

### Demographic characteristics of the patients by sex

The total sample was composed of 2,921 women and 2,605 men. Table 2 shows demographic characteristics of the patients by sex.

**Table 2:** Demographic characteristics of the patients by sex

	Men	Women	p value
	n = 2,605	n = 2,921	
Age; mean (SD)	56.7(11.8)	61.7(12)	< 0.0001
BMI (kg/m^2^); mean (SD)	33.6(4.1)	34.2(3.3)	< 0.0001
Diabetes mellitus; n (%)	773 (29.7)	954 (32.7)	0.02
SAH; n (%)	1.843 (70.7)	2,263 (77.5)	< 0.001
Family history of IHD; n (%)	429 (16.5)	652 (22.3)	< 0.001
Smoking; n (%)	270 (10.4)	196 (6.7)	< 0.001
Dyslipidemia; n (%)	1,369 (52.5)	1,627 (55.7)	0.02
**Symptoms before SPECT-MPI; n (%)**			**< 0.001**
Asymptomatic	1,701 (65.8)	1.295 (45.2)	
Atypical angina	433 (16.7)	777 (27.2)	
Typical angina	108 (4.2)	254 (8.9)	
Tiredness	343 (13.3)	535 (18.7)	
**Stress protocol; n (%)**			**< 0.001**
Physical	1,895 (72.7)	1,681 (57.5)	
Pharmacological	710 (27.3)	1,240 (42.5)	
Physical stress in METs; mean (SD)	8.7 (2.2)	6.8 (2.1)	< 0.0001
%LVEF; mean(DP)	54.1 (18.4)	63.9 (15.5)	0.04
**LVEF; n(SD)**			**< 0.0001**
LVEF > 50%	2,126 (89.4)	2,695 (95.9)	
LVEF 30 - 49%	227 (9.5)	103 (3.7)	
LVEF < 30%	25 (1.0)	13 (0.5)	
Abnormal SPECT-MPI abnormal; n (%)	475 (18.2)	813 (27.8)	< 0.001
Ischemia	436 (16.7)	792 (27.1)	
Ischemia > 10% of the LV	45(1.7)	29 (0.9)	0.017
Fibrosis alone	13 (0.5)	9 (0.3)	
Fibrosis and ischemia	26 (1)	12 (0.4)	

SD: standard deviation; BMI: body mass index; SAH; systemic arterial hypertension; IHD: ischemic heart disease METs: metabolic equivalents; LVEF: post-stress left ventricular ejection fraction; SPECT-MPI: myocardial perfusion imaging with single-photon emission computed tomography; LV: left ventricle

### Distribution of patients by BMI

Most patients (70.2%) were class I obese. Table 3 shows the distribution of the patients by BMI.

**Table 3:** Distribution of patients by body mass index

BMI Classification	30.0 - 34.9 kg/m^2^Class I obesity*	35.0 - 39.9 kg/m^2^Class II obesity*	≥ 40.0 kg/m^2^Class III obesity*
Number (%) of patients	n = 3,880 (70.2%)	n = 1,207 (21.8%)	n = 439 (7.9%)

BMI: body mass index.

* World Health Organization^1^

### Percentage of abnormal perfusion according to the BMI

**Figure 1:**
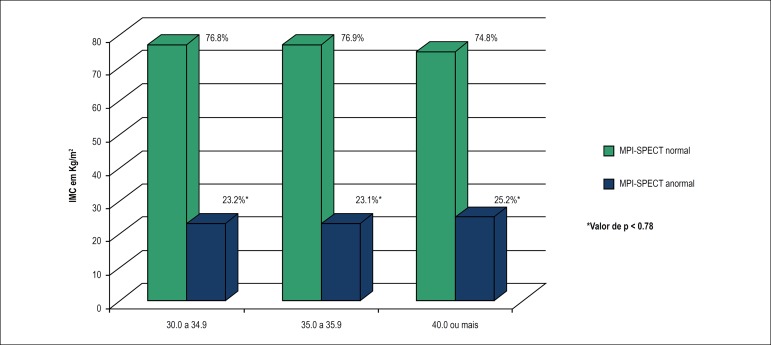
Percentage of abnormal myocardial perfusion imaging with single-photon emission computed tomography (SPECT-MPI) according to BMI in the study population (n = 5,526) *a p-value lower than 0.05 was considered statistically significant.

Among obese individuals (n = 5,526), there was no statistically significant difference in the number of patients with abnormal SPECT-MPI according to BMI. Figure 1 shows the percentage of abnormal SPECT-MPI according to BMI.

### Factors associated with abnormal myocardial perfusion

Univariate analysis revealed that the following factors were associated with abnormal myocardial perfusion - age (OR: 1.04; 95%CI: 1.04-1.05. p < 0.001), female sex (OR: 1.18; 95%CI: 1.18-1.21; p < 0.001), DM (OR: 1.96; 95%CI; 1.72-2.23. p < 0.001); SAH (OR: 1.79; 95%CI: 1.53-2.10; p < 0.001), dyslipidemia (OR: 1.19; 95%CI: 1.04-1.34. p < 0.008), typical angina (OR: 1.96; 95%CI: 1.55-2.48; p < 0.001) or tiredness (OR: 1.29. IC 95%: 1.08-1.54. p = 0.004) before SPECT-MPI, lower stress test duration (mean) (OR: 0.81, 95%CI: 0.78-0.84; p < 0.001) and lower (mean) LVEF (OR: 0.996, 95%CI: 0.993-0.999, *p* <0.041).

After multivariate analysis (Table 4), age, typical angina before SPECT-MPI, need of using the pharmacological stress protocol, less physical exertion (METs), and post-stress LVEF were found to be associated with abnormal myocardial perfusion.

**Table 4:** Factors associated with abnormal prefusion after multivariate analysis in obese patients without known ischemic heart disease (n = 5,526)

	OR (95%CI)	Valor de p
Age; years	1.02 (1.01 - 1.03)	< 0.001
**Body mass index**		
30.0 - 34.9 kg/m^2^	Reference	
35.0 - 39.9 kg/m^2^	0.91 (0.73 - 1.12)	0.38
≥ 40.0 kg/m^2^	0.99 (0.68 - 1.45)	0.97
Male sex	0.82 (0.67 - 1.01)	0.052
Diabetes mellitus	1.57 (1.31 - 1.88)	< 0.001
Systemic arterial hypertension	1.21 (0.98 - 1.50)	0.08
Dyslipidemia	1.14 (0.96 - 1.36)	0.13
**Symptoms before the test**		
Asymptomatic	Reference	
Atypical angina	1.21 (0.97 - 1.49)	0.08
Typical angina	2.45 (1.82 - 3.31)	< 0.001
Tiredness	0.93 (0.72 - 1.20)	0.59
**Stress protocol; n (%)**		
Physical	Reference	
Pharmacological	1.53 (1.18-1.98)	< 0.001
Physical exertion, in METs	0.89 (0.85-0.93)	<0.001
**LVEF**		
LVEF > 50%	Reference	
LVEF 30 - 49%	7.42 (5.3-10.4)	<0.001
LVEF < 30%	10.2 (2.6-40.3)	<0.001

BMI: body mass index, IHD: ischemic heart disease, METs: metabolic equivalents, LVEF: left ventricular ejection fraction. A p < 0.05 was considered statistically significant.

## Discussion

Our study reveals a strong association between obesity and other cardiovascular risk factors. Obesity is known to lead to insulin resistance, SAH, dyslipidemia, thromboembolism and sleep apnea and increase inflammatory markers, all known to be risk factors for CAD.^4^ Obesity is an important factor in the pathogenesis and progression of CAD, with an almost linear relationship between BMI above 25 kg/m^2^ and the risk of CAD.^7^

Xingping et al.^12^ evaluated the relationship between BMI and the prognostic value of SPECT-MPI in 2,096 obese subjects without known CAD (mean age 62 ± 12 years). The authors reported a high prevalence of DM (22%), dyslipidemias (47%) and SAH (61%).^12^ More recently, researchers of The Southern Community Cohort Study investigated the relationship between BMI and late mortality in young adults. At the end of the study, the total sample of obese individuals was 6,276 (mean age 50 ± 7.8 years). In this group, the authors also observed a high prevalence of risk factors - DM in 35.9%, dyslipidemias in 38.8% and SAH in 66.4%.^13,14^

The World Health Organization (WHO) believes that overweight and obesity are responsible for 44% of the risk for DM.^1^ The International Diabetes Federation (IDF) estimates a prevalence of 10-12% of DM among adults in Brazil, which corresponds to 14.5 million people. In addition, the IDF estimates a 60% increase of new cases of DM in Latin America in the next 15 years.^15^

In the DIAD (Detection of Ischemia in Asymptomatic Diabetics) study, the authors assessed whether the screening for CAD could decrease the occurrence of cardiovascular events in symptomatic diabetic patients. A total of 1,123 patients were randomized to screening with SPECT-MPI or to no screening. After a mean follow-up of 4.8 years, the authors did not find any significant differences in cardiovascular event rate between the two groups.^16^ The presence of DM significantly increases cardiovascular risk, and the need for diagnostic methods, including the rational use of nuclear medicine.^17-19^

A significant percentage of our study group (55%) were asymptomatic before the test, especially men. The high proportion of asymptomatic patients in our sample may be explained by several factors, including stratification of future events in patients at high cardiovascular risk, previous altered or inconclusive cardiologic tests, patients referred for preoperative assessment, and the presence of electrocardiographic abnormalities that limit the performance of TT (left branch bundle block, artificial pacemaker rhythm or ventricular preexcitation).^20,21^

Regarding preoperative assessment, the II Guidelines for Perioperative Assessment of the Brazilian Society of Cardiology suggests that indications for SPECT-MPI are similar to those of TT, *i.e*., patients at estimated intermediate risk of vascular surgery complications, without severe cardiovascular conditions in the perioperative period. Also, SPECT-MPI would be the best choice for patients with physical impairment, changes in the ST segment that affect electrocardiography analysis, and in case of possible false positive results in TT.^22^

The decision to screen for IHD among obese patients should be similar to that in the general population, based mainly on clinical symptoms, chest pain and tiredness, and/or the presence of other associated risk factors. Besides, patients’ ability to exercise and the presence of an interpretable electrocardiogram guide us in making decisions about the methods to be used.

Obese subjects are more likely to be screened for IHD, due to the higher presence of associated risk factors, tiredness, low functional capacity and musculoskeletal impairments.^23,24^

In 35% of our patients, a pharmacological stress was used, and this percentage was higher among women than men (42.5% versus 27.3%). This frequency was similar to that reported by Xingping et al.^12^ (38%).

Duvall et al.,^25^ evaluating the prognostic and diagnostic value of SPECT-MPI in 433 morbidly obese patients, observed that 77.4% of the patients used the pharmacological stress protocol, indicating a decreased functional capacity with increase of BMI. The use of pharmacological stress protocols is associated with low functional capacity, non-cardiac physical limitations, low motivation to exercise, left ventricular dysfunction, pulmonary diseases, abnormal electrocardiographic findings at rest (above mentioned), and inappropriate discontinuation of medications prior to the test (e.g., beta-blockers).^20,21^

With respect to demographic differences by gender, most of our patients were women, who showed a more severe cardiovascular risk profile - higher mean BMI, and higher prevalence of associated risk factors (DM, SAH and dyslipidemias). In women, the rates of typical angina were lower, the use of pharmacological stress protocols was more common, and less physical effort during the test compared with men. The percentage of abnormal perfusion in SPECT-MPI was also higher in women than in men (27.8% versus 18.2%).

Studies have shown that women with diagnosis of CAD tend to be older, and present diffuse disease and a worse prognosis than men, including higher acute myocardial infarction and myocardial revascularization surgery. The use of effective diagnostic and prognostic methods, including nuclear medicine, is essential to reduce IHD morbimortality in this group.^18,26^ In a previous study of our group, Cerci et al.,^27^ in a study with 2,250 women, reported a strong, independent association between abnormal SPECT-MPI and mortality among women in Brazil.^27^

In our country, there is little information available about factors associated with abnormalities in myocardial perfusion in obese patients. Our data showed that age, DM, typical angina prior to the test, use of pharmacological stress, less physical effort in the test and lower mean post-stress LVEF were associated with perfusion abnormality. These findings corroborate previous studies on obese and non-obese subjects, with or without previous IHD. In the study by Xingping et al.,^12^ predictive factors of cardiac mortality and abnormal SPECT-MPI in 2,096 obese subjects without known CAD were age, DM, use of pharmacological stress protocol and reduction of LVEF. Greater ability to exercise reduced mortality risk.^14^ Korbee et al.^15^ showed that an abnormal SPECT-MPI, age, and previous heart failure were associated with major cardiovascular events and mortality in obese individuals during up to six years of follow-up following the test. These data have already been included in medical guidelines for appropriate indications of nuclear cardiology in patients with suspected CAD.^28^

If on the one hand obese individuals are at higher risk for CAD, on the other hand, these patients, particularly severely obese subjects, represent a challenging population concerning eligibility to all kinds of cardiac imaging tests.^29,30^

Obesity may affect the quality of SPECT-MPI images, reducing the specificity of the method due to diaphragmatic attenuation or increased extracardiac radiotracer activity. The use of higher doses of radiotracers, attenuation correction techniques, acquisition of images in prone position, among other techniques, may reduce the number of false-positive results related to obesity. Male sex and the use of physical stress protocol by the TT are associated with better quality of the images in obese patients undergoing SPECT-MPI.^27,28^

Positron-emission tomography (PET) with rubidium-82 seems to be the non-invasive method of choice for diagnostic and prognostic assessment of obese individuals with suspected CAD. Sensitivity and specificity of PET with rubidium-82 and SPECT-MPI are estimated to be 91% and 89%, and 87% and 73%, respectively.^31^

Chow et al.,^32^ in a large multicentric study, evaluated the prognostic value (risk of overall and cardiac mortality) in 6,037 patients, 2,016 of them obese. After a mean follow-up period of 2.2 years, the authors concluded that PET with rubidium-82 improved the prognostic estimates of patients of all weight ranges. A normal PET was associated with a very low annual mortality in normal weight (0.38%), overweight (0.43%) or obese (0.15%) subjects.^32^

Although we do not have anatomic information of the patients referred for coronary angiography or coronary angiotomography following SPECT-MPI, we believe that the cases of abnormal SPECT-MPI encompass a wide pathophysiological range, including false-positive cases due to the presence of artifacts, IHD without an obstructive component (associated with endothelial dysfunction or coronary microcirculation impairment), and mostly obstructive CAD.

### Limitations

Our data were systematically collected using a standardized questionnaire administered by a nursing technician, nurses or physicians, and hence, some information regarding clinical variables were self-reported.

Most of patients had not undergone attenuation correction techniques, which help to reduce the percentage of abnormal SPECT-MPI associated with artifacts (false-positive results).

Our study was based on physiological variables and detection of ischemia; thus, we do not have anatomical information of patients that were referred for coronary angiography or coronary angiotomography based on SPECT-MPI results. For this reason, the actual percentage of false-positive cases and abnormal SPECT-MPI associated with obstructive CAD or other IHDs caused by endothelial dysfunction or impaired coronary microcirculation could not be determined.

## Conclusions

Factors associated with abnormal myocardial perfusion in obese patients without known IHD, after adjustment for relevant variables (multivariate analysis) were - age (2% increased risk per year older), DM (57% increased risk in diabetic patients), typical angina (245% increased risk in patients with typical angina as compared with symptomatic patients), use of pharmacological stress during (61% increased risk as compared with physical stress by TT), less physical exertion (expressed in METs) (10% reduced risk for each additional MET during TT) and post-stress LVEF (1% reduced risk for each 1% increase in LVEF).

## References

[1] World Health Organization (2000). Obesity: preventing and managing the global epidemic. Report of a WHO
Consultation.

[2] World Health Organization (2017). 10 facts on obesity.

[3] Sociedade Brasileira de Endocrinologia e Metabologia (2017). http://www.endocrino.org.br/media/uploads/PDFs/vigitel.pdf.

[4] Poirier P, Giles TD, Bray GA, Hong Y, Stern JS, Pi-Sunyer FX (2006). Obesity and cardiovascular disease: pathophysiology, evaluation,
and effect of weight loss: an update of the 1997 American Heart Association
Scientific Statement on Obesity and Heart Disease from the Obesity Committee
of the Council on Nutrition, Physical Activity, and
Metabolism. Circulation.

[5] Poirier P, Eckel RH (2002). Obesity and cardiovascular disease. Curr Atheroscler Rep.

[6] Wormser D, Kaptoge S, Di Angelantonio E, Wood AM, Pennells L, Emerging Risk Factors Collaboration (2011). Separate and combined associations of body-mass index and
abdominal adiposity with cardiovascular disease: collaborative analysis of
58 prospective studies. Lancet.

[7] Rabkin SW, Mathewson FA, Hsu PH (1977). Relation of body weight to development of ischemic heart disease
in a cohort of young North American men after a 26 year observation period
the Manitoba Study. Am J Cardiol.

[8] Manson JE, Colditz GA, Stampfer MJ, Willett WC, Rosner B, Monson RR (1990). A prospective study of obesity and risk of coronary heart disease
in women. N Engl J Med.

[9] Wilson PW, D'Agostino RB, Sullivan L, Parise H, Kannel WB (2002). Overweight and obesity as determinants of cardiovascular risk:
the Framingham experience. Arch Intern Med.

[10] Schinkel AF, Bax JJ, Geleijnse ML, Boersma E, Elhendy A, Roelandt JR (2003). Noninvasive evaluation of ischemic heart disease: myocardial
perfusion imaging or stress echocardiography?. Eur Heart J.

[11] Elhendy A, Schinkel AF, van Domburg RT, Bax JJ, Valkema R, Biagini E (2006). Prognostic stratification of obese patients by 99mTc-tetrofosmin
myocardial perfusion imaging. J Nucl Med.

[12] Xingping K, Shaw LJ, Hayes SW, Hachamovitch R, Abidov A, Cohen I. (2006). Impact of body mass index on cardiac mortality in patients with
know or suspect coronary artery disease undergoing myocardial perfusion
single-photon emission computed tomography. J Am Coll Cardiol.

[13] Korbee RS, Boiten HJ, Ottenhof M, Valkema R, van Domburg RT, Schinkel AF (2013). What is the value of 99mTc-tetrofosmin myocardial perfusion
imaging for the assessment of very long-term outcome in obese
patients?. J Nucl Cardiol.

[14] Hirko KA, Kantor ED, Cohen SS, Blot WJ, Stampfer MJ, Signorello LB (2015). Body mass index in young adulthood, obesity trajectory, and
premature mortality. Am J Epidemiol.

[15] International Diabetes Federation (2017). IDF Diabetes Atlas.

[16] Young LH, Wackers FJ, Chyun DA, Davey JA, Barrett EJ, Taillefer R (2009). Cardiac outcomes after screening for asymptomatic coronary artery
disease in patients with type 2 diabetes: the DIAD study: a randomized
controlled trial. JAMA.

[17] Herman WH, Zimmet P (2012). Type 2 diabetes: an epidemic global requiring global attention
and urgent action. Diabetes Care.

[18] Shaw LJ, Butler J (2014). Targeting priority populations to reduce disparities in
cardiovascular care: health equity for all. J Am Coll Cardiol.

[19] Daviglus ML, Talavera GA, Avilés-Santa ML, Allison M, Cai J, Criqui MH (2012). Prevalence of major cardiovascular risk factors and
cardiovascular diseases among Hispanic/Latino individuals of diverse
backgrounds in the Unites States. JAMA.

[20] Henzlova MJ, Duvall WL, Einstein AJ, Travin MI, Verberne HJ (2016). ASNC imaging guidelines for SPECT nuclear cardiology procedures:
stress, protocols, and tracers. J Nucl Cardiol.

[21] Zaret B, Beller G (2010). Clinical nuclear cardiology: state of the art and future
directions.

[22] Gualandro DM, Yu PC, Caramelli B, Marques AC, Calderaro D, Fornari LS, Sociedade Brasileira de Cardiologia (2017). 3ª Diretriz de avaliação cardiovascular
perioperatória da Sociedade Brasileira de Cardiologia. Arq Bras Cardiol.

[23] Schinkel AF, Bax JJ, Geleijnse ML, Boersma E, Elhendy A, Roelandt JR (2003). Noninvasive evaluation of ischaemic heart disease: myocardial
perfusion imaging or stress echocardiography?. Eur Heart J.

[24] Lim SP, Arasaratnam P, Chow BJ, Beanlands RS, Hessian RC (2015). Obesity and the challenges of noninvasive imaging for the
detection of coronary artery disease. Can J Cardiol.

[25] Duvall WL, Croft LB, Corriel JS, Einstein AJ, Fisher JE, Haynes PS (2006). SPECT myocardial perfusion imaging in morbidly obese patients:
image quality, hemodynamic response to pharmacologic stress, and diagnostic
and prognostic value. J Nucl Cardiol.

[26] Mieres JH, Shaw LJ, Arai A, Budoff MJ, Flamm SD, Hundley WG (2005). Role of noninvasive testing in the clinical evaluation of women
with suspected coronary artery disease consensus statement from the cardiac
imaging committee, council on clinical cardiology, and the cardiovascular
imaging and intervention committee, council on cardiovascular radiology and
intervention, American Heart Association. Circulation.

[27] Cerci MS, Cerci JJ, Cerci RJ, Pereira Neto CC, Trindade E, Delbeke D (2011). Myocardial perfusion imaging is a strong predictor of death in
women. JACC Cardiovasc Imaging.

[28] Hendel RC, Berman DS, Di Carli MF, Heidenreich PA, Henkin RE, Pellikka PA (2009). ACCF/ASNC/ACR/AHA/ASE/SCCT/SCMR/SNM 2009 appropriate use criteria
for cardiac radionuclide imaging: a report of the american college of
cardiology foundation appropriate use criteria task force, the American
Society of Nuclear Cardiology, the American College of Radiology, the
American Heart Association, the American Society of Echocardiography, the
Society of Cardiovascular Computed Tomography, the Society for
Cardiovascular Magnetic Resonance, and the Society of Nuclear
Medicine. J Am Coll Cardiol.

[29] Fiechter M, Gebhard C, Fuchs TA, Ghadri JR, Stehli J, Kazakauskaite E (2012). Cadmium-zinc-telluride myocardial perfusion imaging in obese
patients. J Nucl Med.

[30] Berman DS, Kang X, Nishina H, Slomka PJ, Shaw LJ, Hayes SW (2006). Diagnostic accuracy of gated Tc-99m sestamibi stress myocardial
perfusion SPECT with combined supine and prone acquisitions to detect
coronary artery disease in obese and nonobese patients. J Nucl Cardiol.

[31] Aggarwal NR, Drozdova A, Askew JW 3rd, Kemp BJ, Chareonthaitawee P (2015). Feasibility and diagnostic accuracy of exercise treadmill
nitrogen-13 ammonia PET myocardial perfusion imaging of obese
patients. J Nucl Cardiol.

[32] Chow BJ, Dorbala S, Di Carli MF, Merhige ME, Williams BA, Veledar E (2014). Prognostic value of PET myocardial perfusion imaging in obese
patients. JACC Cardiovasc Imaging.

